# Metabolite-driven protein modifications in immune signaling: from established principles to emerging pyruvylation

**DOI:** 10.3389/fimmu.2026.1912474

**Published:** 2026-08-21

**Authors:** Ziye Zhang, Mingze Xu, Sen Zhang, Zhikun Zhang, Hongguang Zhang, Zhenjie Wang, Min Zhu, Hongchang Zhao

**Affiliations:** 1 Department of Emergency Surgery, The First Affiliated Hospital of Bengbu Medical University, Bengbu, Anhui, China; 2 Department of Clinical Laboratory, The First Affiliated Hospital of Bengbu Medical University, Bengbu, Anhui, China; 3 Institute of Emergency and Critical Care Medicine, The First Affiliated Hospital of Bengbu Medical University, Bengbu, Anhui, China; 4 School of Life Sciences, Anhui Agricultural University, Hefei, Anhui, China

**Keywords:** acetylation, crotonylation, immunometabolism, immunopharmacology, lactylation, pyruvylation

## Abstract

**Background:**

Immune signaling is tightly coupled to cellular metabolic state. Beyond supplying energy, metabolites can directly regulate immune responses by driving post-translational modifications of proteins and chromatin, shifting immunometabolism toward a model in which metabolic state encodes signaling outputs.

**Findings:**

Protein pyruvylation has recently emerged as a metabolite-responsive lysine modification linking glycolytic metabolism to both immune signaling and transcriptional regulation. Established examples, including histone lactylation and acylation marks linked to acetyl-CoA and crotonyl-CoA, illustrate how metabolite availability shapes chromatin state and transcriptional competence. A recent Cell study showed that high glucose-enhanced glycolysis and pyruvate kinase M2 activity promotes STAT1 pyruvylation at Lys201, thereby disrupting STAT1-STAT2 interaction and suppressing type I interferon signaling. Complementing this signaling-centered mechanism, a subsequent Nature Metabolism study systematically characterized a broader lysine pyruvylation landscape, identified histone and non-histone substrates, linked pyruvylation to glycolytic flux and pyruvyl-CoA metabolism, and implicated HAT1 and p300 as pyruvylation writers and SIRT3 as an eraser. Together, these findings expand pyruvylation from a single signaling event into an emerging metabolite-responsive regulatory system operating across protein signaling and chromatin-associated transcriptional control. In this review, we summarize the conceptual framework of metabolite-driven protein modifications, compare established marks, and discuss the remaining questions surrounding pyruvylation chemistry, enzyme and substrate specificity, reader mechanisms, compartmentalization, detection strategies, physiological relevance, and potential immunopharmacological implications.

**Conclusions:**

Metabolite-driven protein modifications represent an important regulatory layer linking metabolic rewiring to immune reprogramming. Elucidating the chemistry, regulatory machinery, substrate landscape, and physiological functions of pyruvylation will not only advance our understanding of immunometabolism but may also facilitate the development of metabolite-based biomarkers and therapeutic strategies for inflammatory and immune-related disease.

## 1 Introduction

Immune responses are inseparable from cellular metabolic state (1–3). Activated macrophages, dendritic cells, and other innate immune populations undergo rapid metabolic rewiring characterized by increased glycolysis, altered tricarboxylic acid cycle activity, and context-dependent changes in oxidative phosphorylation (4, 5). These metabolic adaptations were initially understood as bioenergetic solutions that support cell survival, cytokine production, and biosynthetic demand (6). However, a broader view has emerged in which metabolites are not merely passive products or substrates, but active regulators of signaling and gene expression (7, 8).

A major advance in this field has been the recognition that metabolites can influence immune output by driving post-translational modifications (PTMs) of proteins (9). The discovery of histone lactylation provided a compelling demonstration that lactate, long regarded as a glycolytic by-product, can directly serve as a precursor for a chromatin-associated modification that stimulates gene transcription (10–12). Likewise, acetylation and crotonylation have reinforced the principle that metabolite pools are tightly linked to epigenetic and transcriptional control (13, 14). Together, these findings have transformed immunometabolism from a discipline focused mainly on pathway utilization into one concerned with how metabolic state is chemically translated into regulatory information (15).

This conceptual shift has recently been extended by two complementary studies of protein pyruvylation. In a 2026 Cell study (16), pyruvate was shown to covalently modify STAT1 at Lys201, generating a pyruvylated form of STAT1 that impairs STAT1-STAT2 association and attenuates type I interferon signaling. The same study further demonstrated that high glucose, glycolytic activity, and PKM2 promote this modification, suggesting that pyruvate may function as a direct regulator of immune signaling beyond its established metabolic roles (16). More recently, an independent Nature Metabolism study substantially broadened this framework by identifying a wider spectrum of histone and non-histone pyruvylated proteins, linking global pyruvylation to glycolytic flux and pyruvyl-CoA metabolism, and providing evidence that HAT1 and p300 promote lysine pyruvylation whereas SIRT3 functions as a depyruvylase (17). Genome-wide profiling further associated pyruvylation with promoter regions and transcriptional activity (17). Together, these studies suggest that pyruvylation may operate at multiple regulatory levels, ranging from direct remodeling of immune signaling complexes to chromatin-associated transcriptional control.

## 2 A conceptual framework for metabolite-driven protein modifications

### 2.1 Metabolic rewiring: more than bioenergetics

Metabolic rewiring is now recognized as a hallmark of immune activation (18, 19). Upon stimulation by microbial products or inflammatory cytokines, innate immune cells rapidly shift toward aerobic glycolysis (20–22), while changes in mitochondrial function and intermediary metabolism help sustain inflammatory or regulatory programs (19, 23). This rewiring does more than provide ATP. It reshapes the intracellular abundance, localization, and flux of metabolic intermediates, thereby creating biochemical conditions that can influence signaling proteins and chromatin regulators (24).

Importantly, the functional consequences of this rewiring cannot always be explained by energy balance alone. Similar receptor inputs can produce distinct immune outputs depending on glucose availability (25), mitochondrial fitness (26, 27), or metabolite accumulation (28). This observation has led to a more integrated model in which metabolism influences immunity at multiple layers, including transcription factor activity (29), chromatin accessibility (30), signaling complex assembly (31), and stress adaptation (32, 33).

A representative example illustrating this principle is the effect of glucose availability on innate immune responses. Under high-glucose conditions, enhanced glycolytic flux increases intracellular pyruvate and lactate production (34), which can reshape cytokine expression and inflammatory signaling through metabolite-driven PTMs (35, 36). Conversely, glucose restriction or glycolytic inhibition suppresses metabolite accumulation and attenuates downstream inflammatory responses (37, 38). These observations demonstrate that identical immune stimuli may generate distinct signaling outputs depending on the underlying metabolic state, providing experimental support for the general model described above.

**Figure 1:**
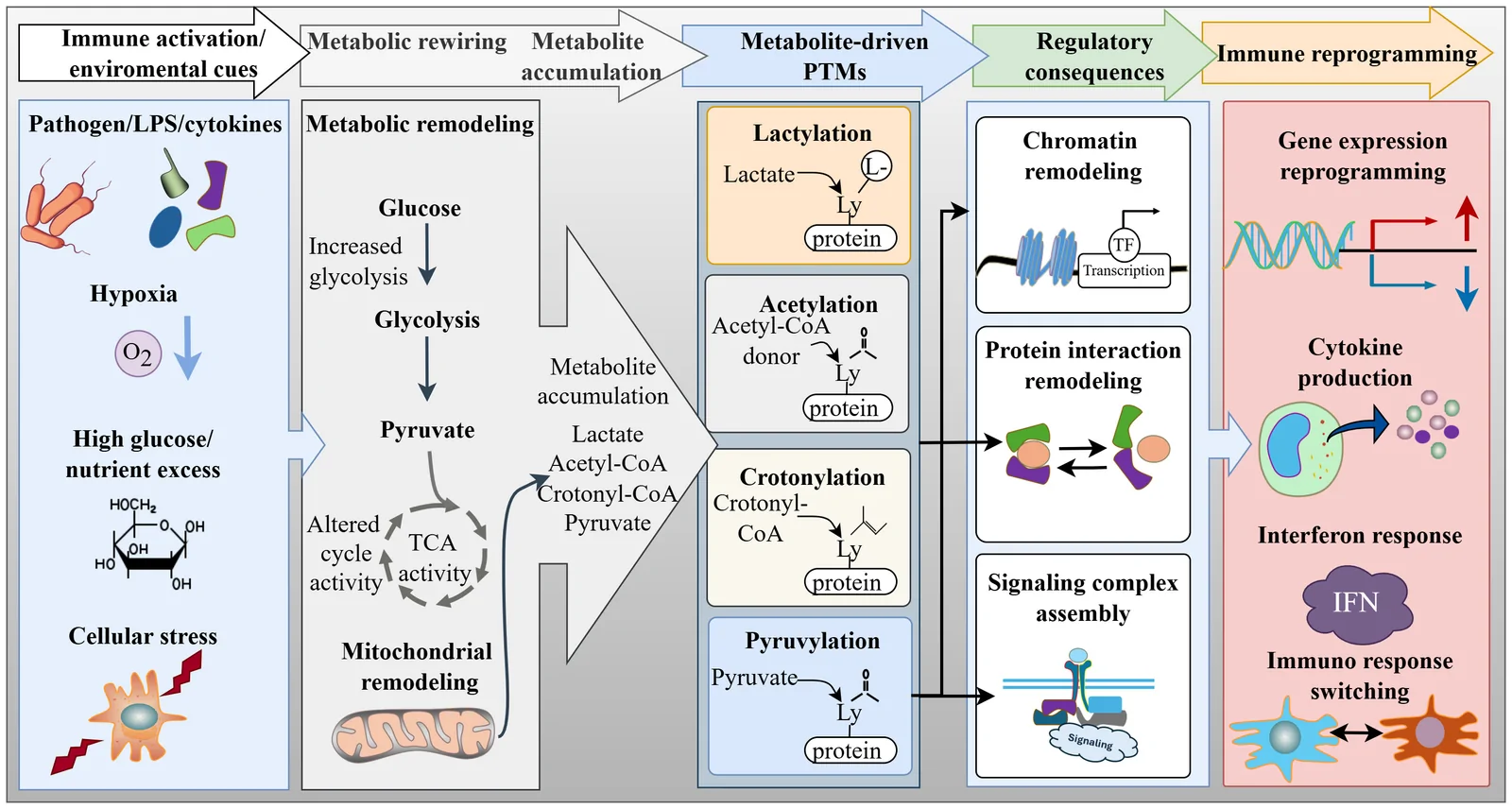
Conceptual framework of metabolite-driven post-translational modifications in immune signaling. Immune activation and environmental cues, including pathogen-associated stimuli, hypoxia, nutrient excess, and cellular stress, induce metabolic rewiring characterized by enhanced glycolysis, altered tricarboxylic acid cycle activity, mitochondrial remodeling, and metabolite accumulation. These metabolic changes promote the generation of metabolite-driven post-translational modifications (PTMs), including lactylation, acetylation, crotonylation, and pyruvylation. In turn, these PTMs reshape immune signaling by regulating chromatin remodeling, protein-protein interactions, and signaling complex assembly, ultimately leading to immune reprogramming at the levels of gene expression, cytokine production, interferon response, and immune response switching.

In this model, metabolites are not only determinants of cellular capacity but also determinants of signaling interpretation (**Figure 1**).

### 2.2 Metabolites as donors or drivers of protein modification

One of the clearest mechanisms by which metabolites can shape immune behavior is through the generation of PTMs (39, 40). In some cases, a metabolite or a metabolite-derived donor provides the chemical group that is transferred onto lysine or other amino acid residues (41, 42). In other cases, metabolite abundance shifts the balance of enzymatic activity, substrate competition, or compartmentalized cofactor supply, thereby indirectly promoting or suppressing modification (43). Acetyl-CoA, crotonyl-CoA, and lactate-associated pathways exemplify this principle, linking central carbon metabolism to protein and histone modifications that alter transcriptional output (44–48).

This view helps explain why metabolic perturbations so often exert selective effects on immune signaling. Rather than acting solely through bulk energetic stress, metabolites can impose a chemical “annotation” on regulatory proteins and chromatin. The resulting PTM landscape may then determine whether inflammatory genes (49, 50), interferon-stimulated genes (51), or repair-associated programs are preferentially expressed (52). As more metabolite-linked PTMs are identified, it is becoming increasingly plausible that immune cells use metabolite availability as a way to encode environmental state into molecular signaling architecture (53).

Pyruvylation occupies a distinctive position within metabolite-driven PTMs. Initial evidence from STAT1 supported pyruvate as a metabolic determinant of lysine pyruvylation, whereas more recent work has implicated pyruvyl-CoA as an activated donor associated with broader lysine pyruvylation (17). The identification of HAT1 and p300 as pyruvylation writers and SIRT3 as a depyruvylase further suggests that at least a subset of pyruvylation events can be enzymatically regulated (17). Nevertheless, the precise relationship between free pyruvate, pyruvyl-CoA generation, non-enzymatic chemistry, and enzyme-catalyzed transfer remains incompletely resolved. Thus, rather than asking whether pyruvylation is enzymatically regulated at all, a central question is which biochemical routes dominate in different cellular compartments and physiological contexts.

### 2.3 How metabolite-driven PTMs reshape immune signaling

Metabolite-driven PTMs can influence immune signaling through several non-exclusive mechanisms (39, 54). First, they can alter chromatin structure and transcriptional competence, thereby changing the accessibility or activity of immune gene loci (55). Second, they can modify signaling proteins directly, affecting stability, localization, conformational state, or interaction with partner proteins (56, 57). Third, they may influence the timing and amplitude of signal transduction by controlling the formation of higher-order complexes rather than simply changing phosphorylation abundance (58).

This framework is especially useful because it accommodates both established and emerging examples. Histone lactylation exemplifies a gene-regulatory mechanism linked to glycolytic lactate production, whereas pyruvylation appears to act at the level of protein-protein interaction within interferon signaling. Taken together, these findings suggest that metabolite-driven PTMs are not a narrow epigenetic curiosity but a broader organizing principle through which metabolic context reprograms immune output.

## 3 Established metabolite-linked modifications in immune regulation

### 3.1 Lactylation

Lactylation is among the most influential examples of a metabolite-driven post-translational modification (PTM) entering mainstream immunology (59). In a landmark 2019 study, Zhang and colleagues showed that lysine lactylation stimulates gene transcription, establishing lactate as a direct contributor to epigenetic regulation rather than merely a glycolytic by-product. In activated macrophages, bacterial stimulation and hypoxia enhanced glycolytic lactate production, which was accompanied by increased histone lactylation and activation of genes associated with inflammatory resolution (59). This discovery fundamentally reshaped the field of immunometabolism by demonstrating that metabolic end product can be chemically translated into transcriptional regulation (60).

From a metabolic perspective, lactylation provides an elegant example of how immune cell metabolism is coupled to gene regulation. During innate immune activation, macrophages and other immune cells rapidly increase aerobic glycolysis, leading to pyruvate accumulation (61). Pyruvate is subsequently converted into lactate by lactate dehydrogenase (LDH), resulting in intracellular lactate accumulation (62). Rather than representing metabolic waste, elevated lactate now appears to serve as a signaling precursor capable of driving lysine lactylation, thereby linking glycolytic flux directly to transcriptional control (63). This metabolic axis illustrates a broader principle that cellular metabolism not only fuels immune responses but also encodes regulatory information through metabolite-derived protein modifications.

Subsequent studies have considerably expanded the biological scope of lactylation. Beyond histones, numerous non-histone proteins have been identified as lactylation substrates, indicating that lactylation regulates multiple layers of immune signaling (64). In macrophages, histone lactylation promotes the transition from pro-inflammatory activation toward tissue repair and inflammatory resolution (65). In adaptive immunity, emerging evidence suggests that lactylation participates in T-cell differentiation, immune tolerance, and cytokine regulation (66). In addition, lactylation has been implicated in dendritic cell activation, tumor-associated macrophage polarization, and chronic inflammatory remodeling, highlighting its diverse immunological functions across both innate and adaptive immune systems (67).

Mechanistically, substantial progress has also been made in identifying the enzymatic machinery responsible for lactylation. Recent evidence suggests that HBO1 functions as a histone lactyltransferase capable of catalyzing histone lactylation under physiological conditions (68). Other lysine acetyltransferases, including p300, have also been proposed to contribute to lactylation under specific experimental settings, although their precise substrate specificity and physiological relevance remain under active investigation (69). Importantly, lactylation is a reversible modification. Multiple members of the histone deacetylase (HDAC) family, particularly HDAC1, HDAC2, and HDAC3, together with SIRT2, have been reported to exhibit delactylase activity, providing an enzymatic mechanism for dynamic regulation of lactylation during immune activation (70, 71). The identification of both writers and erasers indicates that lactylation is not simply a passive consequence of metabolite accumulation but rather a tightly regulated signaling process.

The physiological significance of lactylation has now been documented in numerous disease settings. In sepsis, elevated lactate levels and histone lactylation have been linked to immune reprogramming and inflammatory resolution (72). In cancer, excessive glycolysis and lactate accumulation within the tumor microenvironment promote widespread lactylation events that contribute to immune suppression, macrophage polarization, and tumor progression (73, 74). Lactylation has also been implicated in tissue fibrosis, autoimmune disorders, chronic infection, and metabolic diseases, emphasizing that lactate-dependent protein modification represents a common regulatory mechanism across multiple pathological contexts (60, 75, 76).

Taken together, lactylation has evolved from a single epigenetic observation into the prototype of metabolite-driven immune regulation (76). More importantly, it established a conceptual framework demonstrating that metabolites are capable of functioning as signaling molecules by generating chemically distinct PTMs that regulate chromatin organization, protein function, and immune cell behavior. This paradigm paved the way for the discovery of additional metabolite-driven PTMs and provides the conceptual foundation upon which newly identified modifications such as pyruvylation can now be interpreted.

### 3.2 Acetylation

Acetylation represents one of the best-characterized examples of how metabolic intermediates regulate gene expression and immune function through post-translational modification. Unlike lactylation, which is primarily linked to glycolytic lactate accumulation, lysine acetylation is directly coupled to intracellular acetyl-CoA availability, making acetyl-CoA not only a central metabolic intermediate but also an essential substrate for epigenetic and protein regulation (47, 77–79). Consequently, fluctuations in acetyl-CoA production, subcellular compartmentalization, and metabolic flux can directly influence the extent of protein acetylation and downstream immune responses (80, 81).

Multiple metabolic pathways contribute to the intracellular acetyl-CoA pool, including glucose oxidation through pyruvate dehydrogenase, ATP citrate lyase (ACLY)-mediated conversion of citrate, and acetate recycling through acetyl-CoA synthetase 2 (ACSS2) (13, 82, 83). These interconnected pathways enable immune cells to dynamically couple nutrient availability with transcriptional regulation (84). During immune activation, metabolic remodeling alters acetyl-CoA abundance (85), thereby influencing the activity of lysine acetyltransferases (KATs), including p300/CBP and GCN5, which catalyze histone and non-histone acetylation (86, 87). Conversely, histone deacetylases (HDACs) and sirtuins function as major deacetylases, establishing acetylation as a highly dynamic and reversible modification responsive to metabolic changes.

Although histones remain the best-studied substrates, acetylation extends far beyond chromatin regulation. Numerous transcription factors and signaling molecules involved in innate and adaptive immunity are regulated by lysine acetylation (88). For example, acetylation modulates the activity of NF-κB, STAT proteins, p53, and FOXO family members, thereby influencing inflammatory gene expression, cytokine production, cellular differentiation, and stress responses (89–92). These observations indicate that acetylation functions not only as an epigenetic regulator but also as a direct modulator of intracellular signaling networks.

In immune cells, acetylation has emerged as an important mechanism linking metabolic state to cell fate determination (93, 94). Changes in acetyl-CoA metabolism influence macrophage activation, dendritic cell maturation, T-cell differentiation, and memory T-cell formation by coordinating chromatin accessibility with transcriptional programs (66, 94, 95). In addition, aberrant acetylation has been implicated in chronic inflammation, autoimmune diseases, infection, and tumor immunity, highlighting its broad physiological and pathological significance (96–98). Mechanistically, because acetylation is supported by a well-defined enzymatic system comprising established writers, erasers, and readers, it serves as a mature paradigm illustrating how metabolic intermediates can regulate immune responses through reversible protein modification (99).

Overall, acetylation demonstrates a central principle that extends across metabolite-driven PTMs: metabolic intermediates are not merely substrates for energy production but actively participate in immune regulation by shaping protein function and transcriptional outputs. Compared with emerging metabolite-driven modifications such as pyruvylation, acetylation represents a highly evolved regulatory network with extensive mechanistic characterization, thereby providing an important conceptual benchmark for understanding newly identified metabolite-dependent signaling mechanisms.

### 3.3 Crotonylation

Crotonylation further expands the repertoire of metabolite-driven post-translational modifications by illustrating how short-chain acyl-CoA metabolites can generate functionally distinct regulatory signals. Histone lysine crotonylation (Kcr) was first identified in 2011 by Tan and colleagues as a conserved histone modification that is structurally and functionally distinct from lysine acetylation. Although both modifications utilize lysine residues as acceptor sites, the planar crotonyl group contains a rigid carbon-carbon double bond, conferring greater hydrophobicity and steric characteristics that can differentially influence chromatin architecture, protein recognition, and transcriptional activation (100).

Unlike acetylation, which depends on acetyl-CoA, crotonylation is driven by intracellular crotonyl-CoA availability (101). Crotonyl-CoA can be generated through multiple metabolic pathways, including fatty acid β-oxidation, lysine and branched-chain amino acid catabolism, and short-chain fatty acid metabolism (45). Consequently, fluctuations in nutrient utilization and mitochondrial metabolism directly influence the intracellular crotonyl-CoA pool, thereby coupling metabolic state to chromatin regulation (102). Similar to other acyl modifications, crotonylation is dynamically regulated by dedicated enzymatic machinery (103). Histone acetyltransferases such as p300 have been shown to possess crotonyltransferase activity, whereas several HDAC family members and sirtuins exhibit decrotonylase activity (104–107). In addition, YEATS-domain-containing proteins preferentially recognize crotonylated lysine residues, providing a specialized reader system that distinguishes crotonylation from other lysine acylations (108).

Functionally, crotonylation is strongly associated with transcriptionally active chromatin and has emerged as an important regulator of immune gene expression (109). Genome-wide studies have demonstrated that histone crotonylation preferentially marks active promoters and enhancers, where it facilitates transcriptional activation of genes involved in inflammation, cellular differentiation, and host defense (106, 110). Compared with acetylation, crotonylation often exhibits stronger transcriptional activation at selected loci, suggesting that individual metabolite-derived acyl groups encode distinct regulatory information rather than serving as interchangeable chemical variants (111).

Increasing evidence indicates that crotonylation contributes to immune regulation across multiple physiological and pathological contexts (39, 112). In macrophages, alterations in crotonylation accompany inflammatory activation and metabolic reprogramming (113, 114). Emerging studies also suggest roles for crotonylation in regulating innate immune signaling, antiviral responses, and inflammatory transcriptional programs (45, 115), although these mechanisms remain less extensively characterized than those governing acetylation. In addition, dysregulated crotonylation has been associated with cancer, inflammatory disorders, and tissue injury (112, 116), further supporting the concept that metabolite-derived acyl modifications participate broadly in immune adaptation to metabolic stress.

In summary, crotonylation demonstrates that subtle chemical differences among metabolite-derived acyl groups can generate distinct biological outcomes. Rather than representing a simple biochemical variant of acetylation, crotonylation possesses unique metabolic regulation, dedicated reader proteins, and specialized transcriptional functions. Together with lactylation and acetylation, it provides compelling evidence that metabolite-derived PTMs constitute a diverse regulatory language through which cellular metabolism is translated into immune signaling and gene regulation.

### 3.4 Shared principles and distinct features of metabolite-driven PTMs

Although lactylation, acetylation, crotonylation, and pyruvylation differ in their chemical donors, enzymatic regulation, and biological targets, they collectively illustrate a common conceptual framework whereby metabolic intermediates are translated into regulatory information through covalent protein modification. Rather than acting solely as substrates for energy production or biosynthesis, metabolites function as signaling molecules capable of encoding cellular metabolic status into chromatin remodeling, protein activity, and immune signaling (117). This emerging paradigm represents a fundamental shift from the traditional view that metabolism merely supports immune activation to the concept that metabolism directly instructs immune cell behavior (118).

A shared feature of these metabolite-driven PTMs is their intimate dependence on metabolic flux (117, 119). Alterations in glycolysis, mitochondrial oxidative metabolism, fatty acid oxidation, or amino acid catabolism reshape intracellular metabolite availability (120), thereby influencing the abundance of acetyl-CoA, crotonyl-CoA, lactate, or pyruvate (81, 121). Changes in these metabolite pools are subsequently translated into dynamic PTM landscapes through dedicated or emerging enzymatic systems, allowing immune cells to integrate nutrient availability with transcriptional and signaling outputs. In this sense, metabolite-driven PTMs serve as molecular interfaces connecting metabolic adaptation to immune regulation (122, 123).

Despite these common principles, important mechanistic differences distinguish individual modifications. Acetylation and crotonylation are supported by well-characterized writers, erasers, and reader proteins and primarily regulate chromatin accessibility and transcriptional competence (124). Lactylation likewise exerts major epigenetic functions but also extends to non-histone proteins, thereby broadening the regulatory influence of glycolytic metabolism (125). Pyruvylation remains an emerging PTM but has rapidly progressed beyond its initial identification on STAT1. Current evidence now supports at least two regulatory dimensions: at the signaling level, STAT1 K201 pyruvylation directly disrupts STAT1-STAT2 complex formation and attenuates type I interferon signaling (16); at the chromatin-associated level, broader lysine pyruvylation has been detected on histone and non-histone substrates and associated with promoter occupancy and transcriptional activity (17). These findings suggest that pyruvylation, like other metabolite-driven PTMs, may operate across multiple hierarchical levels rather than being restricted to either chromatin regulation or cytosolic signaling.

Viewed together, these observations indicate that metabolite-driven PTMs constitute a diverse yet interconnected regulatory language. Individual metabolites generate chemically distinct modifications that differ in substrate preference, enzymatic control, temporal dynamics, and biological function, yet all converge on a shared objective: translating metabolic state into coordinated immune responses. Understanding both the common organizational principles and the unique biological properties of individual metabolite-driven PTMs will be essential for interpreting recently discovered modifications such as pyruvylation and for identifying general mechanisms through which metabolism shapes innate and adaptive immunity.

The molecular characteristics, regulatory machineries, and current knowledge gaps of representative metabolite-driven PTMs are summarized in **Tables 1**, **2**.

**Table 1:** Established and emerging metabolite-linked protein modifications in immune regulation.

Feature	Lactylation	Acetylation	Crotonylation	Pyruvylation
Metabolite source	Lactate	Acetyl-CoA	Crotonyl-CoA	Pyruvate/pyruvyl-CoA
Representative substrates	Histones; non-histone proteins (11)	Histones; transcription factors; signaling proteins (126, 127)	Histones; chromatin-associated proteins (104)	STAT1 (Lys201); histones; multiple non-histone proteins (16, 17)
Primary regulatory level	Chromatin remodeling and transcriptional activation (11, 128)	Chromatin regulation and transcriptional control; non-histone protein regulation (129, 130)	Active chromatin regulation and transcriptional competence (105, 131)	Signaling complex assembly; chromatin-associated transcriptional regulation (16, 17)
Representative immune consequence	Links glycolytic flux to gene activation and inflammatory program resolution (132, 133)	Regulates immune gene expression, inflammatory signaling, and cellular activation state (92, 115)	Supports transcription-associated immune regulatory programs (134)	Suppresses type I interferon signaling through STAT1 K201; broader immune functions remain to be defined (16, 17)
Mechanistic mode	Metabolite-associated modification linked to lactate accumulation (135)	Acyl-donor-dependent lysine modification linked to acetyl-CoA availability (136, 137)	Acyl-donor-dependent lysine modification linked to crotonyl-CoA availability (112, 138)	Metabolically responsive lysine modification linked to glycolytic flux and pyruvate/pyruvyl-CoA availability
Representative signaling output	Gene expression reprogramming (59)	Transcriptional competence and immune gene regulation (130, 139)	Chromatin activation-associated transcriptional output (105)	Reduced ISG transcription; broader transcriptional regulation (16, 17)
Representative or proposed disease contexts	Sepsis, cancer, fibrosis, infection (39, 73, 115, 132, 140)	Autoimmunity, cancer, inflammation (139, 141)	Cancer, inflammatory diseases (142, 143)	Viral infection and hyperglycemia-associated immune dysfunction (16); cancer-associated dysregulation (17); sepsis (proposed)
Evidence status	Established	Established	Established	Emerging, with independent mechanistic and proteome-level support (16, 17, 144)

This table summarizes representative metabolite-linked protein modifications, including lactylation, acetylation, crotonylation, and pyruvylation, with respect to their metabolite sources, representative substrates, primary regulatory levels, immune consequences, mechanistic features, and current evidence status. Together, these modifications illustrate how metabolic intermediates and end products can be translated into distinct regulatory signals that shape immune transcription, signaling, and functional reprogramming. Evidence status was classified according to the availability of independent mechanistic validation and reproducibility across multiple studies.

**Table 2:** Molecular characteristics of representative metabolite-driven protein modifications.

Feature	Lactylation	Acetylation	Crotonylation	Pyruvylation
Metabolite donor	Lactate	Acetyl-CoA	Crotonyl-CoA	Pyruvate-associated; pyruvyl-CoA implicated as an activated donor
Major metabolic source	Glycolysis → Lactate	Glycolysis/TCA cycle; ACLY; ACSS2	Fatty acid β-oxidation; lysine and BCAA catabolism	Glycolysis; pyruvate/pyruvyl-CoA metabolism; PKM2 activity
Representative writers	HBO1; p300 (context-dependent)	p300/CBP, GCN5, PCAF	p300/CBP, MOF (reported)	HAT1; p300/EP300
Representative erasers	HDAC1/2/3; SIRT2	HDACs; Sirtuins	HDAC1/2/3; SIRT1/2/3	SIRT3
Representative readers	Emerging	Bromodomain proteins	YEATS-domain proteins	Unknown
Major substrates	Histones; non-histone proteins	Histones; TFs; signaling proteins	Histones; chromatin proteins	STAT1 (Lys201); histones; multiple non-histone proteins
Primary regulatory mechanism	Chromatin remodeling; transcription	Chromatin remodeling; protein regulation	Active chromatin regulation	Signaling complex regulation; chromatin-associated transcriptional control
Representative immune functions	Resolution of inflammation; macrophage polarization	Immune gene expression; T-cell differentiation	Immune transcriptional activation	IFN-I suppression; broader immune functions remain to be established
Detection approaches	Pan-Kla antibody; LC-MS/MS	Anti-acetyllysine antibody; LC-MS/MS	Pan-Kcr antibody; LC-MS/MS	Pan-Kpy and site-specific antibodies; LC-MS/MS; diagnostic ions; targeted enrichment; CUT&Tag
Current evidence level	Extensive	Extensive	Moderate	Emerging/rapidly expanding
Major knowledge gaps	Precise writer specificity	Cell-type specificity	Functional diversity	Donor biogenesis; enzyme specificity; readers; compartmentalization; site-specific functions; disease relevance

*Note: Commercial antibodies recognizing protein pyruvylation have recently become available, although their specificity and performance in different biological contexts require continued validation.

Collectively, these established metabolite-driven PTMs demonstrate that metabolites regulate immunity through multiple complementary mechanisms, ranging from chromatin remodeling to direct modulation of signaling proteins. Despite this progress, important conceptual and technical limitations remain before these modifications can be fully integrated into a unified framework of immunometabolism.

### 3.5 Unresolved questions and limitations of established metabolite-linked PTMs

Despite substantial progress, several unresolved issues limit how we interpret established metabolite-linked PTMs such as lactylation, acetylation, and crotonylation in immune regulation. A central challenge is cell-type and state specificity. The same modification can be associated with distinct, or even opposing, transcriptional and functional outcomes depending on cellular lineage, activation state, and microenvironmental context (122). This likely reflects differences in metabolic wiring, compartmentalized metabolite pools, expression of writers/erasers/readers, and chromatin accessibility across immune cell types (122, 145, 146).

A second limitation concerns context-dependent circuit logic in complex disease settings. In heterogeneous systems such as tumors, metabolic gradients (e.g., hypoxia, nutrient depletion, lactate/pyruvate accumulation) coexist with diverse immune and stromal cell populations, making it difficult to define causal regulatory loops linking a specific metabolite to a specific PTM and to downstream immune phenotypes (147–149). Even when associations are robust *in vitro*, mapping the directionality of regulation *in vivo* remains challenging because metabolite availability and PTM dynamics are shaped by spatial heterogeneity, temporal fluctuations, and intercellular metabolic crosstalk (147, 150, 151).

Third, *in vivo* validation and quantitative interpretation remain nontrivial (152). Many studies rely on bulk tissue measurements or ex vivo stimulation, which can obscure cell-specific modification patterns and fail to capture rapid turnover or compartment-specific regulation (153, 154). Moreover, metabolic heterogeneity introduces substantial variability in the magnitude and timing of PTM deposition, complicating cross-study comparability and mechanistic inference (155). Addressing these issues will likely require integrated approaches combining cell-resolved metabolomics, quantitative PTM proteomics, spatial profiling, and genetic perturbation strategies in physiologically relevant models (156).

Another important technical challenge concerns preservation of endogenous metabolite-driven PTMs during sample preparation. Because free intracellular metabolites remain abundant immediately after cell lysis, artificial non-enzymatic modifications may occur during protein extraction unless metabolic activity is rapidly quenched. Future studies should optimize rapid metabolic quenching, low-temperature extraction, and chemical stabilization strategies to preserve endogenous pyruvylation and lactylation states.

Together, these limitations highlight a broader point: metabolite-linked PTMs form a regulatory layer whose interpretation depends critically on cellular context, disease architecture, and metabolic heterogeneity. This perspective is particularly relevant when evaluating emerging modifications such as pyruvylation, where establishing cell-type specificity, *in vivo* causality, and quantitative dynamics will be essential for moving from conceptual novelty to generalizable principles.

Having established the common principles governing metabolite-driven PTMs, we next discuss pyruvylation as the newest member of this regulatory family.

## 4 Protein pyruvylation: from a STAT1 signaling modification to an emerging metabolite-responsive PTM system

Compared with established metabolite-driven PTMs, pyruvylation remains newly recognized but is now supported by complementary site-specific, proteome-wide, enzymatic, and chromatin-level evidence.

### 4.1 Why pyruvylation matters

Pyruvate has long been recognized as the terminal product of glycolysis and a key metabolic hub linking cytosolic glucose catabolism to mitochondrial oxidation and lactate metabolism (157–159). Yet despite its centrality, its non-metabolic functions have remained poorly defined. The proposal that pyruvate can directly participate in a protein post-translational modification is biologically important, as it suggests that one of the most canonical metabolites in intermediary metabolism may also act as a signaling modifier.

This matters for at least two reasons. First, pyruvate occupies a uniquely strategic metabolic position, meaning that its abundance can change rapidly under high-glucose or high-glycolysis conditions. Second, if pyruvate can directly modify signaling proteins, then metabolic flux itself may be able to rewrite immune responsiveness without necessarily changing receptor abundance or upstream phosphorylation cascades. In that sense, pyruvylation is not only a recently discovered PTM, but also a new way to conceptualize how glycolytic state is translated into immune function.

### 4.2 Molecular evidence for STAT1 pyruvylation

A pioneering Cell study provides the first site-specific mechanistic evidence for protein pyruvylation by identifying STAT1 as a pyruvate-modified protein (16). In that work, biotin-pyruvate bound STAT1, and mutation of Lys201 markedly reduced this interaction. The investigators then generated a STAT1 Lys201 pyruvylation-specific antibody and showed that endogenous STAT1 undergoes pyruvylation. Based on quantitative mass spectrometry combined with pyruvylation-specific enrichment, the authors estimated that approximately 30% of endogenous STAT1 molecules were pyruvylated under high-glucose conditions (16).

Additional support came from metabolic and biochemical manipulation. Exogenous pyruvate increased STAT1 pyruvylation in a dose-dependent manner, blockade of pyruvate entry into mitochondria enhanced the modification, and an *in vitro* assay showed that recombinant STAT1 could be modified by pyruvate at Lys201 (16). High glucose also promoted STAT1 pyruvylation, whereas 2-deoxyglucose suppressed it, linking the modification to glycolytic activity (16). Together, these findings provide substantial initial evidence for STAT1 pyruvylation as a metabolically regulated PTM.

### 4.3 Expansion of the pyruvylation landscape: from STAT1 to the pyruvylome

The identification of STAT1 K201 pyruvylation provided the first signaling-centered framework for understanding how pyruvate may directly regulate protein function. More recent work has substantially expanded this view by demonstrating that lysine pyruvylation is not restricted to a single signaling protein. Systematic proteomic analysis identified 88 pyruvylated lysine sites across histone and non-histone proteins, supporting the existence of a broader cellular pyruvylome (17). Importantly, global pyruvylation responded dynamically to intracellular pyruvate availability, glucose concentration, glycolytic inhibition, hypoxia, and altered mitochondrial pyruvate utilization, providing independent evidence that this modification is metabolically regulated (17).

This study further detected endogenous pyruvyl-CoA and identified HAT1 and p300 as pyruvylation writers, while SIRT3 displayed depyruvylase activity (17). Genome-wide CUT&Tag profiling revealed preferential enrichment of histone pyruvylation at promoter regions and a positive association with transcriptional activity. Functional analyses further indicated that changes in glycolytic flux and pyruvylation can influence expression of selected genes, supporting a role for pyruvylation in coupling central carbon metabolism to chromatin-associated transcriptional regulation (17).

Taken together, the two recent studies define complementary dimensions of pyruvylation biology. STAT1 K201 pyruvylation demonstrates site-specific control of immune signaling with *in vivo* functional consequences, whereas pyruvylome and chromatin analyses support a broader metabolite-responsive modification system. This convergence substantially strengthens the evidence for pyruvylation as a bona fide emerging PTM while raising new questions regarding donor utilization, enzyme specificity, substrate selectivity, and context-dependent biological functions.

### 4.4 Conceptual significance of pyruvate-mediated protein modification

The conceptual significance of this work extends beyond STAT1 itself. These findings suggest that pyruvate can function as a small-molecule protein modifier, thereby expanding its biological repertoire beyond energy metabolism. This possibility reframes pyruvate from a central intermediate in carbon metabolism into a potential determinant of signaling specificity under conditions of metabolic stress.

Equally important, the initial STAT1 pyruvylation study highlights a mechanism distinct from the dominant chromatin-centered view of metabolite-linked regulation. Here, the principal effect is not presented as histone remodeling, but as modification of a signaling protein in a way that changes its molecular interactions. This broadens the emerging field of metabolite-driven PTMs and suggests that direct regulation of signaling complexes may become a major theme in immunometabolism.

### 4.5 Pyruvylation in the broader landscape of metabolite-driven PTMs

Pyruvylation was initially defined through a single well-characterized signaling substrate, but the emerging pyruvylome now broadens its conceptual and mechanistic scope. When viewed alongside lactylation, acetylation, and crotonylation. Current evidence indicates that pyruvylation can couple metabolic state to both protein interaction networks and chromatin-associated transcriptional programs (16, 17).

The distinction between pyruvylation and established acylations is therefore no longer a simple contrast between signaling proteins and chromatin. Instead, the available studies suggest a dual-layer model in which site-specific pyruvylation can directly alter signaling complex assembly, while broader histone and non-histone pyruvylation may influence transcriptional regulation (16, 17). Different metabolite-driven PTMs may thus share regulatory levels but differ in substrate preference, enzyme usage, kinetics, stoichiometry, and context-dependent functional weight.

Beyond differences in substrate repertoire, metabolite-driven PTMs also differ in their physicochemical properties. Acetylation generally neutralizes the positive charge of lysine residues (160), whereas crotonylation introduces a bulkier and more rigid unsaturated acyl group that may alter local structural conformation (161). Lactylation contributes an additional hydroxyl-containing side chain that may influence hydrogen-bonding interactions. By comparison, the structural consequences of pyruvylation remain largely undefined. Although pyruvate contains a carbonyl group that could potentially modify steric properties and local electrostatic environments, its precise impact on protein conformation, binding affinity, and signaling complex assembly has not yet been experimentally determined (16). Resolving these structural consequences represents an important future direction for understanding how pyruvylation regulates immune signaling.

The emerging substrate landscape also challenges the initial view that pyruvylation may primarily target cytosolic signaling proteins. The identification of both histone and non-histone substrates suggests that pyruvylation can operate across subcellular compartments and regulatory layers (17). A key unresolved question is therefore not whether pyruvylation is restricted to non-histone proteins, but how donor availability, writer localization, substrate accessibility, and eraser activity determine compartment-specific pyruvylation patterns.

Importantly, the discovery of pyruvylation raises the broader possibility that additional metabolite-driven PTMs remain to be identified. Numerous central metabolites generated during glycolysis, the tricarboxylic acid cycle, amino acid metabolism, and lipid metabolism undergo substantial fluctuations during immune activation (140, 162–164). Whether these metabolites can similarly generate regulatory protein modifications represents an important question for future investigation. From this perspective, pyruvylation may represent not the endpoint of metabolite-driven PTM discovery, but rather the beginning of a broader expansion of the immunometabolism field.

## 5 The high glucose-glycolysis-PKM2-pyruvate axis regulates STAT1 pyruvylation

**Figure 2:**
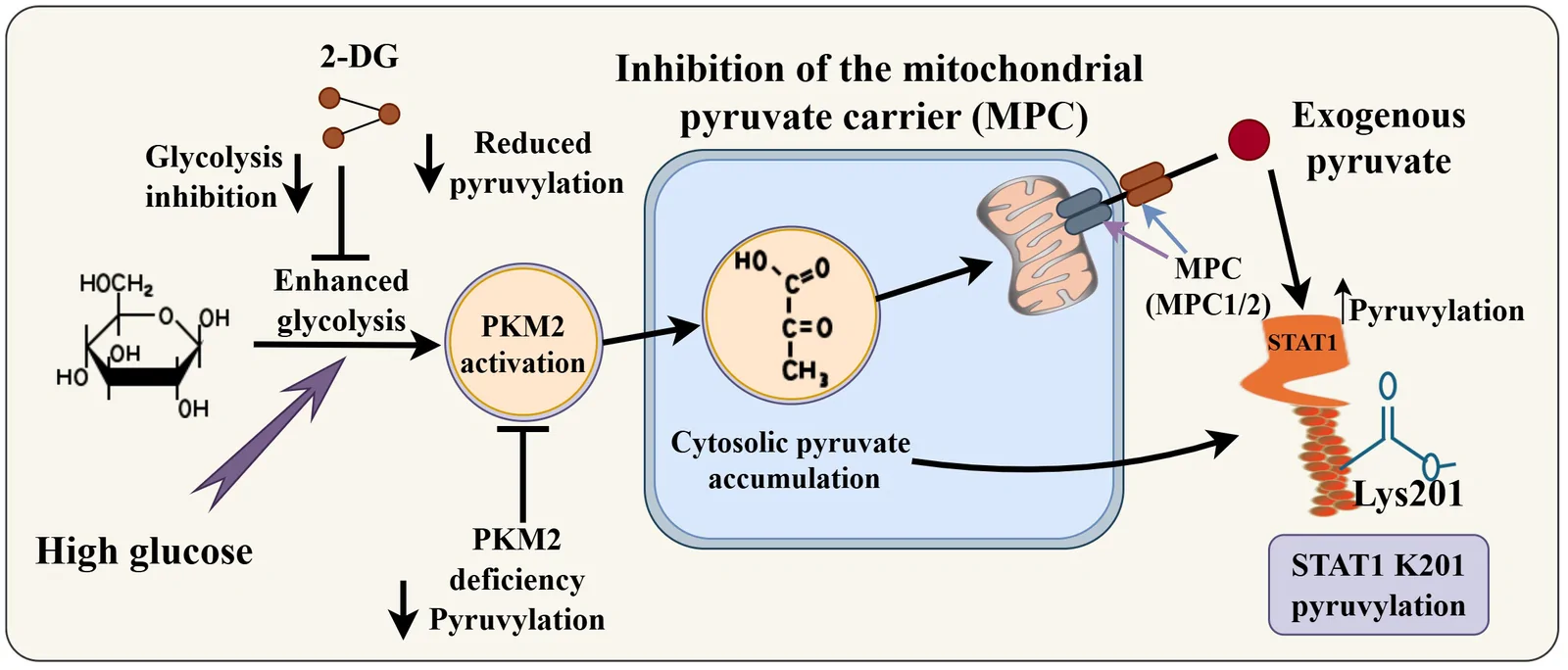
Metabolic regulation of STAT1 pyruvylation. High glucose conditions enhance glycolytic flux and promote PKM2-dependent pyruvate production, thereby increasing the cytosolic pyruvate pool. Inhibition of glycolysis by 2-deoxyglucose suppresses pyruvylation, whereas exogenous pyruvate supplementation promotes it. Inhibition of the mitochondrial pyruvate carrier (MPC) further increases cytosolic pyruvate availability and favors STAT1 pyruvylation. Together, these findings support a model in which metabolic context directly controls STAT1 K201 pyruvylation through the high glucose-glycolysis-PKM2-pyruvate axis.

A major strength of the pyruvylation study is that it did not stop at modification discovery, but traced the upstream metabolic logic that drives this event (16). The data support a pathway in which high glucose enhances glycolysis, increases pyruvate availability, and thereby promotes STAT1 pyruvylation. Exogenous pyruvate was sufficient to increase STAT1 pyruvylation, whereas 2-deoxyglucose suppressed it. In addition, blocking pyruvate import into mitochondria increased cytosolic pyruvate and further enhanced the modification (**Figure 2**) (16).

PKM2 emerges as a particularly important upstream node in this axis. As a glycolytic enzyme that catalyzes phosphoenolpyruvate-to-pyruvate conversion, PKM2 is well positioned to influence pyruvate supply (165). In the Cell study, PKM2 promoted pyruvylation-related signaling effects, and PKM2 deficiency weakened the pathway (16). This observation is notable because it links a well-known metabolic regulator to an emerging PTM, thereby reinforcing the idea that metabolic enzyme activity can influence immune signaling not only through pathway flux but also through downstream chemical modification of signaling proteins.

The broader implication is that pyruvylation is unlikely to be a constitutive or context-free modification. Instead, it appears to behave as a metabolically sensitive modification whose abundance depends on nutrient state, glycolytic flux, and intracellular pyruvate handling. This makes pyruvylation particularly relevant to conditions characterized by glucose excess, inflammatory metabolic rewiring, or impaired mitochondrial pyruvate handling.

PKM2 is well known to undergo dynamic transitions between highly active tetrameric and less active dimeric forms, with the latter also participating in non-metabolic nuclear functions. Whether STAT1 pyruvylation is primarily driven by global cytosolic pyruvate production resulting from tetrameric PKM2 activity or by localized pyruvate microenvironments associated with dimeric PKM2 remains unknown. Current evidence supports PKM2 as an upstream determinant of pyruvate availability but does not distinguish between these mechanistic possibilities. Future studies integrating metabolic flux analysis, spatial metabolomics, and high-resolution proteomics will be required to determine whether pyruvylation is governed by global metabolic flux or localized metabolic microdomains.

The identification of pyruvyl-CoA in the broader pyruvylation pathway adds another layer to this metabolic model (17). Rather than viewing free pyruvate abundance as the sole determinant of modification, current evidence suggests that pyruvate may feed into distinct biochemical routes: direct or locally facilitated modification of selected signaling substrates such as STAT1, and activated-donor-dependent enzymatic pyruvylation involving pyruvyl-CoA and acyltransferases such as HAT1 or p300 (16, 17). Whether these routes coexist within the same cells, operate in distinct subcellular compartments, or preferentially target different substrate classes remains an important question.

## 6 How pyruvylation reshapes immune signaling

One of the most intriguing features of STAT1 pyruvylation is the level at which it acts. The available data indicate that pyruvate did not primarily suppress interferon signaling by reducing receptor abundance or preventing canonical phosphorylation events. Instead, pyruvylation altered the ability of STAT1 to interact with STAT2 (16). This places the functional consequence of the modification at the level of signaling complex assembly rather than simple on/off phosphorylation control.

Mechanistically, this is important because it suggests that metabolite-driven PTMs may fine-tune signaling by changing the topology of protein interaction networks. In the pyruvylation study, STAT1 Lys201 modification weakened STAT1-STAT2 association, suppressed interferon-stimulated gene expression such as Ifit1 and Isg15, and reduced antiviral defense. Conversely, STAT1-K201R knock-in mice displayed stronger type I interferon-mediated antiviral responses, supporting the *in vivo* relevance of this modification (16). From the structure perspective, STAT1 Lys201 is located within the coiled-coil domain of STAT1, a region implicated in intermolecular interactions and signaling complex assembly. Although pyruvylation at Lys201 markedly disrupts STAT1–STAT2 complex formation, the precise structural mechanism underlying this effect remains unknown. It is possible that modification at this site alters local conformational dynamics or interaction surfaces required for complex assembly; however, this hypothesis awaits future structural studies using approaches such as cryo-electron microscopy or molecular dynamics simulations.

**Figure 3:**
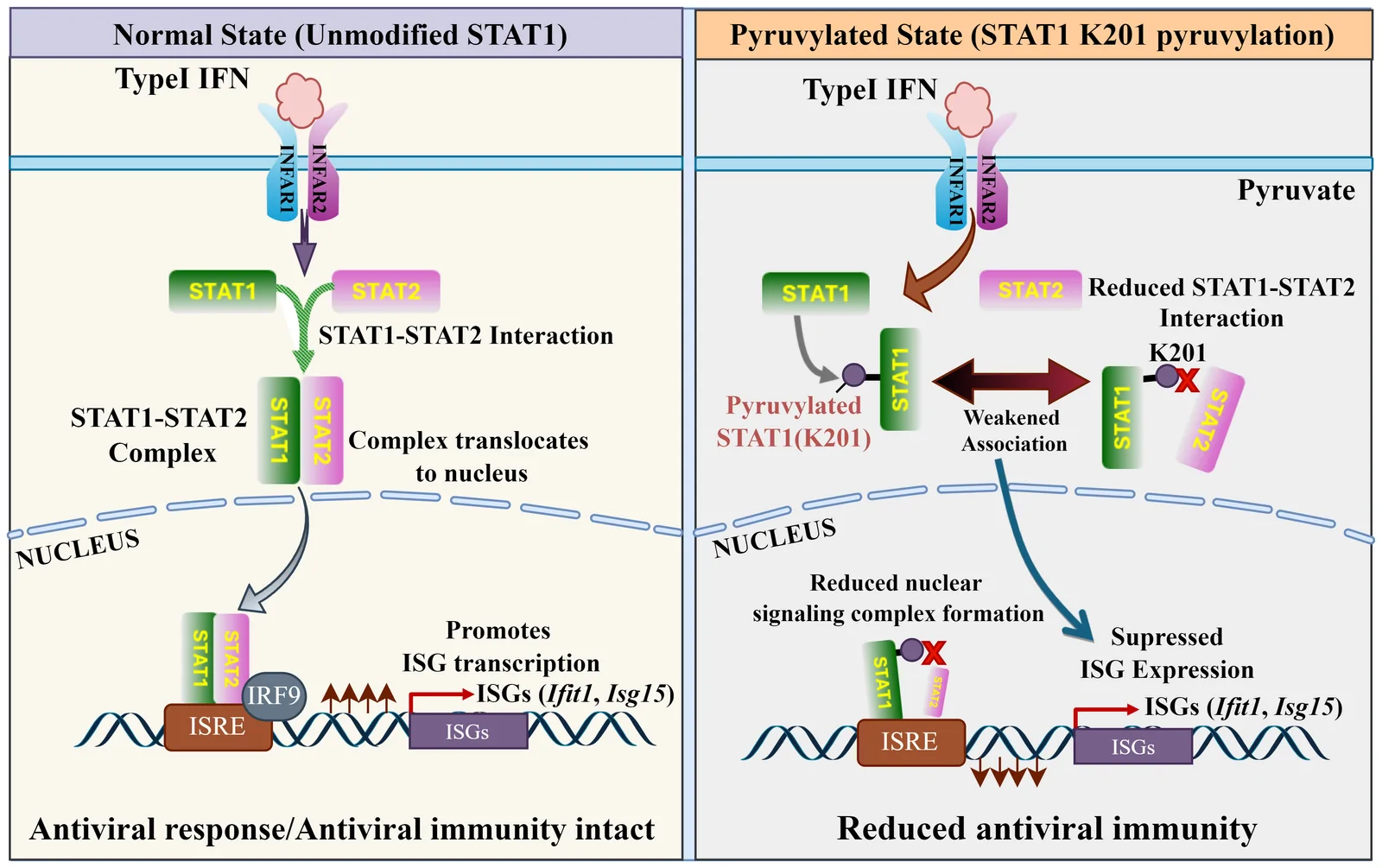
STAT1 pyruvylation rewires type I interferon signaling by disrupting STAT1-STAT2 complex formation. Under normal conditions, type I interferon signaling promotes STAT1-STAT2 complex formation and induces interferon-stimulated gene (ISG) expression. Pyruvylation of STAT1 at Lys201 impairs STAT1-STAT2 interaction without primarily altering upstream receptor abundance or canonical STAT1 phosphorylation. As a result, ISG transcription is reduced, leading to suppression of type I interferon signaling and weakened antiviral immunity. This mechanism highlights how a metabolite-driven PTM can regulate immune output at the level of signaling complex assembly.

More broadly, this mechanism suggests that metabolic context may modulate signal output even when phosphorylation appears intact. In other words, metabolite-driven PTMs may act as “fine-tuners” that reprogram immune output by reshaping signal interpretation downstream of receptor activation (**Figure 3**).

These mechanistic insights raise a broader question: does pyruvylation represent an isolated phenomenon or a general principle of metabolic immune regulation?

## 7 Toward a general principle of metabolite-driven immune regulation

The convergence of site-specific mechanistic evidence from STAT1 with broader proteomic and chromatin-level characterization now provides a stronger basis for considering pyruvylation as part of a general metabolite-responsive regulatory system (16, 17). Current evidence supports a dual-layer model of pyruvylation biology: a signaling layer, in which site-specific modification directly remodels protein interactions, and a transcriptional layer, in which broader lysine pyruvylation couples glycolytic state to chromatin-associated gene regulation. In this view, metabolite abundance is not simply a background variable but a biochemical parameter that can be written onto proteins in the form of PTMs, thereby influencing signaling complex assembly and transcriptional output.

Viewed from this perspective, PTMs act as critical intermediates between metabolic stress and immune phenotype. Cells experiencing high glycolytic flux, nutrient overload, or altered mitochondrial handling of metabolites may generate a PTM landscape distinct from that of metabolically quiescent cells. The resulting differences in chromatin state and signaling architecture may help explain why identical inflammatory or interferon inputs can produce divergent outcomes under different metabolic states. The clinical relevance of this metabolic coding is perhaps most profound in the context of sepsis-induced immune collapse and the tumor microenvironment (TME).

In sepsis, acute hyperglycemic and enhanced glycolytic flux may increase intracellular pyruvate availability, raising the possibility that STAT1 pyruvylation could occur under these conditions. Although this mechanism has not yet been experimentally validated in sepsis models, we propose that excessive pyruvylation may contribute to impaired type I interferon signaling and immune dysfunction. Future experimental studies will be required to determine whether this conceptual model operates *in vivo*. Similarly, in the TME, the Warburg Effect creates a chronic state of metabolite accumulation, imposing a repressive PTM landscape on infiltrating immune cells. Notably, accumulating evidence indicates that metabolite-driven PTMs can directly regulate innate immune signaling beyond chromatin remodeling. For example, lactylation has been reported to modulate the cGAS–STING pathway by influencing cGAS activity and stability, illustrating that metabolite-derived protein modifications can regulate signaling proteins in addition to histones. These findings provide an important conceptual precedent for pyruvylation, suggesting that metabolite-driven PTMs may control immune responses through multiple hierarchical mechanisms, ranging from transcriptional regulation to direct remodeling of signaling complex assembly. Building on this conceptual framework, we propose that pyruvylation may represent a distinct and perhaps more glucose-sensitive regulatory layer.

**Figure 4:**
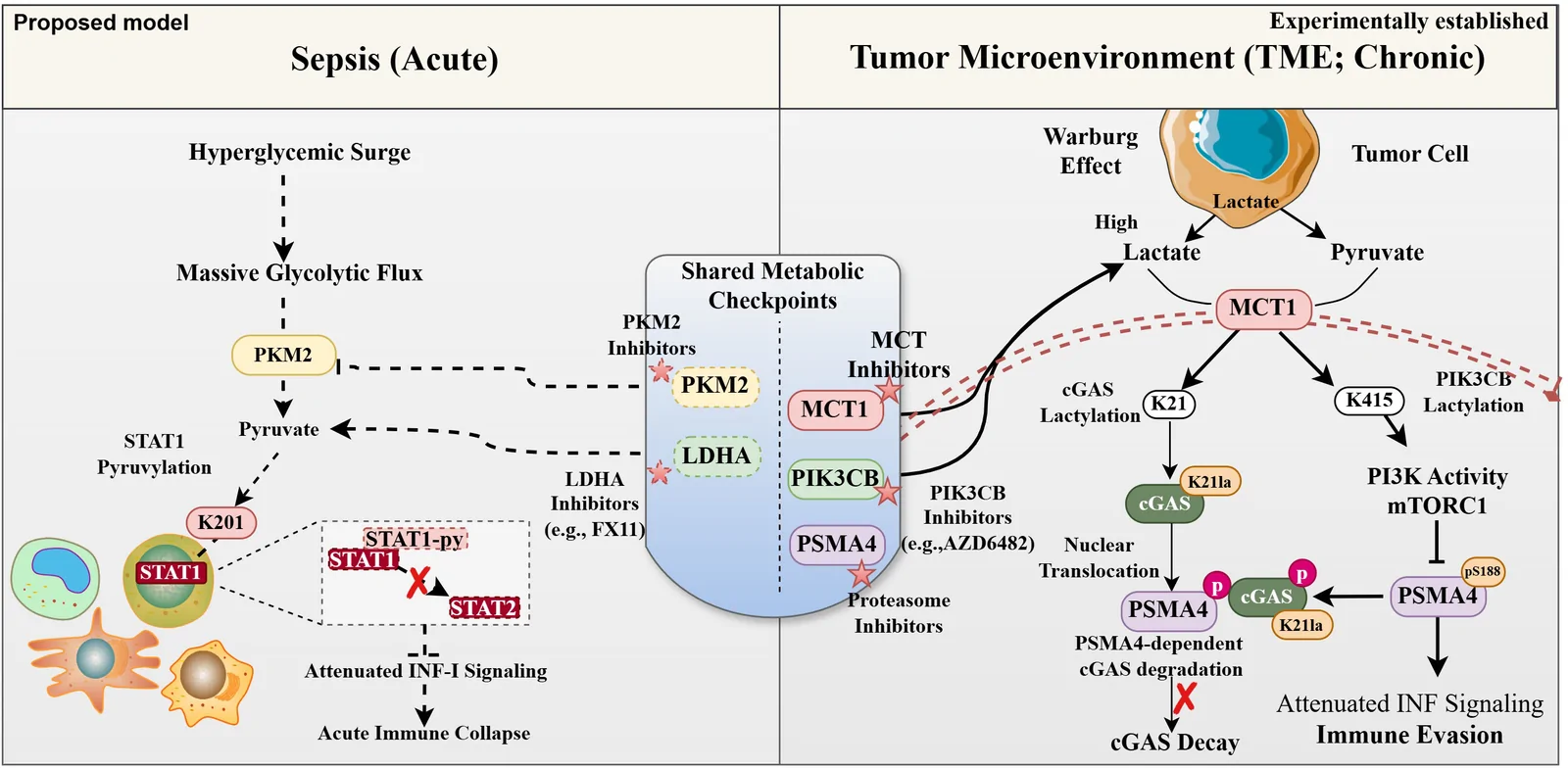
Disease-specific models and therapeutic implications of metabolite-driven PTMs. The left panel illustrates a hypothesis proposed in this Review, in which acute hyperglycemia, enhanced glycolysis, and increased pyruvate availability may promote STAT1 pyruvylation and contribute to impaired type I interferon signaling during sepsis. This model has not yet been experimentally validated in sepsis and is intended as a conceptual framework. The right panel summarizes experimentally established mechanisms reported in the literature, including lactylation-mediated regulation of the cGAS-STING pathway and PIK3CB signaling in the tumor microenvironment. Solid arrows indicate experimentally supported molecular mechanisms. Dashed arrows indicate proposed biological models or theoretical extrapolations developed in this Review.

**Figure 4** distinguishes experimentally established lactylation pathways from the conceptual pyruvylation model proposed in this Review, thereby highlighting both current evidence and future hypotheses. More broadly, pyruvylation strengthens the case for a modification-centered view of immunometabolism.

Rather than asking only which pathways are upregulated or suppressed, future studies may increasingly ask how metabolic states are chemically inscribed onto signaling proteins and chromatin. In this sense, pyruvylation may ultimately matter as much for the principle it reveals as for the specific STAT1 example that first exposed it.

## 8 Current challenges and future directions

Despite its conceptual appeal, pyruvylation remains an early-stage field with several major unresolved issues.

First, the biochemical routes leading to pyruvylation require further clarification. Recent evidence implicates pyruvyl-CoA as an activated donor for enzymatic lysine pyruvylation, whereas the STAT1 study demonstrates a close dependence on free pyruvate availability (16, 17). Determining the relative contributions of free pyruvate, pyruvyl-CoA-dependent enzymatic transfer, locally facilitated reactions, and potentially non-enzymatic chemistry will be essential for defining the molecular basis of distinct pyruvylation events.

If pyruvylation involves a reversible Schiff-base intermediate or related carbonyl chemistry, chemical trapping strategies such as sodium borohydride (NaBH4) reduction may provide useful experimental approaches for stabilizing transient intermediates. However, because the precise chemical nature of the pyruvylation linkage remains unresolved, these approaches should be interpreted cautiously and combined with orthogonal structural analyses, including high-resolution mass spectrometry, isotope tracing, and NMR or cryo-EM where appropriate.

Second, the enzymatic machinery requires broader validation and mechanistic resolution. HAT1 and p300 have been implicated as pyruvylation writers and SIRT3 as an eraser, representing a major advance over the initial state of the field (17). However, their substrate specificity, catalytic efficiency, cellular compartmentalization, and relative contributions across different cell types remain incompletely understood. Whether additional dedicated writers, erasers, or reader proteins exist is also unknown.

Third, the central challenge has shifted from determining whether additional pyruvylated proteins exist to identifying which of the newly mapped sites are functionally consequential. Proteome-scale identification establishes substrate breadth but does not by itself demonstrate biological function. Site-specific genetic editing, rescue experiments, structural analyses, and quantitative stoichiometric measurements will therefore be required to distinguish regulatory pyruvylation events from low-abundance or functionally neutral modifications. Particular attention should be given to immune signaling proteins, chromatin regulators, and metabolic enzymes whose activities depend on protein-protein interactions or lysine accessibility.

Fourth, translational opportunities are promising but premature. If pyruvylated STAT1 or other pyruvylated proteins can be robustly detected in accessible clinical specimens (e.g., PBMCs), they may enable biomarker development for metabolic suppression of immune signaling and facilitate patient stratification. From an immunopharmacology perspective, the most tractable route may ultimately lie in the regulatory machinery of pyruvylation. If dedicated writers, erasers, and readers are identified, they could offer targets to modulate immune signaling with greater specificity than broadly altering metabolism—by inhibiting a writer, enhancing or mimicking an eraser, or blocking a reader–substrate interaction to uncouple the modification from downstream consequences. Robust assays and quantitative mapping will be essential to support target validation, compound screening, and biomarker-guided clinical translation.

**Figure 5:**
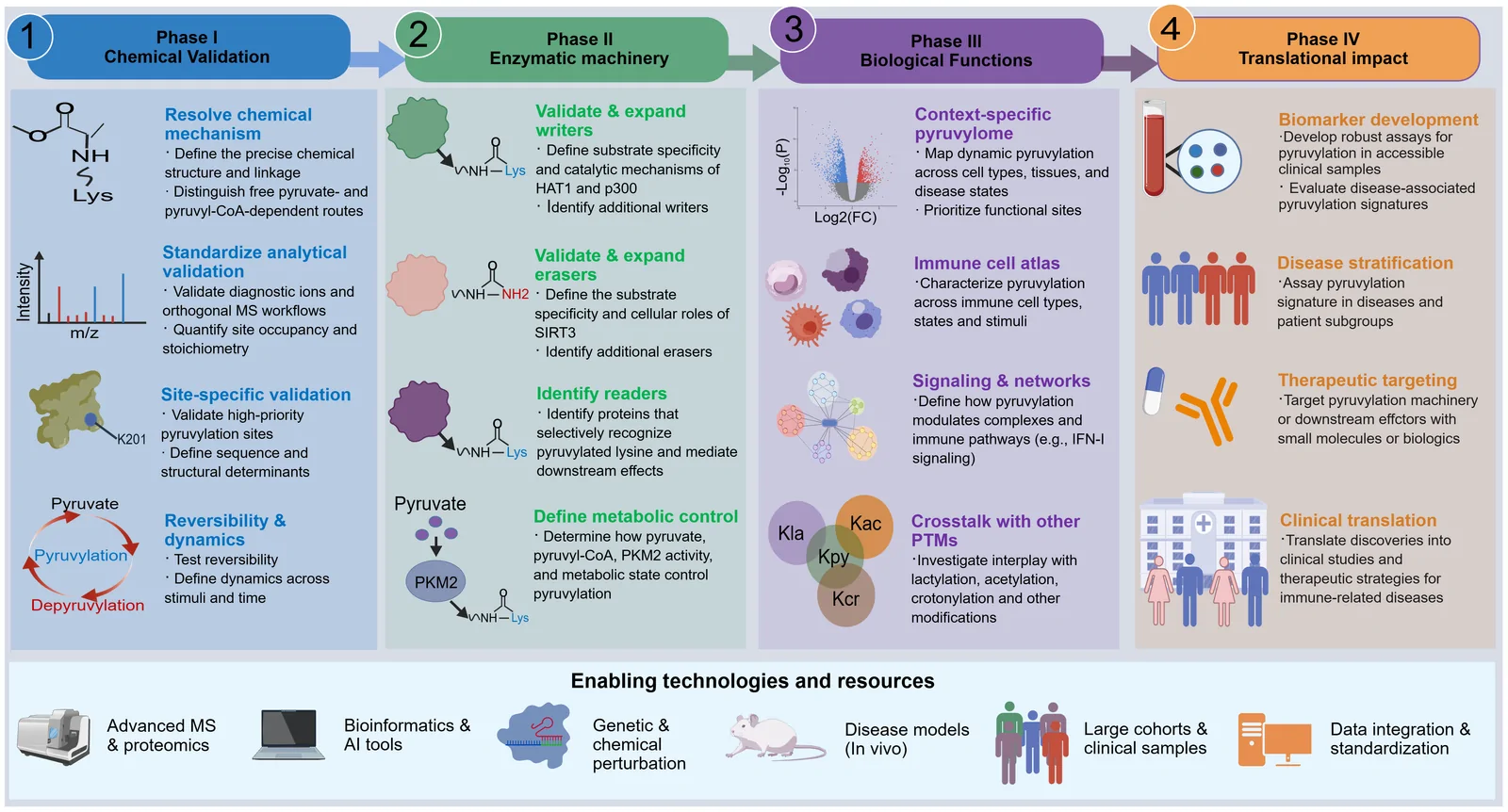
Future roadmap for pyruvylation research. An updated roadmap for advancing the pyruvylation field from biochemical definition to translational application. First, rigorous chemical studies should resolve the relative roles of free pyruvate, pyruvyl-CoA, and distinct reaction routes. Second, the enzymatic architecture should be validated and expanded by defining HAT1/p300 substrate specificity, SIRT3-dependent removal, additional writers or erasers, reader proteins, and pyruvyl-CoA biogenesis. Third, the existing pyruvylome should be expanded and functionally annotated across immune cell types, metabolic states, subcellular compartments, and disease models, with site-specific validation and analysis of cross-talk with other lysine acylations. Finally, translation into clinical applications may enable biomarker development, disease stratification, and therapeutic targeting of pyruvylation-associated pathways.

The key priorities for future pyruvylation research are summarized in **Figure 5**. These efforts will be essential to establish pyruvylation as a bona fide regulatory modification and to determine its translational value in immune-related diseases.

Finally, methodological standardization remains important despite rapid technical progress. Pan-Kpy and site-specific antibodies, characteristic diagnostic ions, LC-MS/MS workflows, targeted enrichment, and CUT&Tag-based chromatin profiling now provide complementary approaches for pyruvylation detection (16, 17). Continued validation of antibody specificity, quantitative performance, site localization, and cross-platform reproducibility will be necessary for robust comparison across biological systems.

Future pyruvylome studies should now move beyond initial mapping toward cell-type-specific, stimulus-dependent, and spatially resolved functional annotation. Priority systems include type I interferon signaling, the cGAS-STING pathway, pattern-recognition receptor networks, chromatin regulators, and metabolic enzymes. Integrating genetic perturbation with quantitative proteomics, spatial metabolomics, and transcriptional profiling will help identify high-confidence sites whose modification is both metabolically regulated and functionally necessary.

Addressing these challenges will determine whether pyruvylation evolves from an intriguing biochemical observation into a broadly conserved mechanism of immune regulation.

## 9 Conclusions

Collectively, current evidence supports a model in which metabolic intermediates function not only as metabolic substrates but also as direct regulators of immune signaling and transcription through chemically distinct PTMs. Lactylation, acetylation, and crotonylation have established that metabolic state can shape gene expression and immune identity through covalent modification. The recent emergence of pyruvylation further extends this framework. Site-specific studies of STAT1 K201 demonstrate that pyruvylation can remodel signaling complex assembly and suppress type I interferon responses, whereas complementary proteomic and chromatin-level analyses reveal a broader lysine pyruvylation landscape linked to glycolytic flux, pyruvyl-CoA metabolism, HAT1/p300-dependent writing, SIRT3-mediated removal, and transcriptional regulation (16, 17). Together, these findings move pyruvylation beyond a single-protein observation and support its recognition as an emerging metabolite-responsive regulatory system.

Although lactylation and pyruvylation both illustrate how glycolytic metabolites are translated into regulatory signals, their current evidence bases and functional emphases differ. Lactylation is supported by extensive studies across chromatin remodeling, non-histone protein regulation, and immune cell reprogramming. Pyruvylation is less mature but now spans direct control of signaling protein interactions and promoter-associated transcriptional regulation. These complementary mechanisms show that metabolite-driven PTMs can influence immune function at both epigenetic and signaling-complex levels. Defining how pyruvylation interfaces with lactylation, acetylation, and crotonylation may reveal competition, cooperation, or substrate switching among metabolite-sensitive lysine modifications.

Future studies should now move beyond establishing the existence of pyruvylation toward defining its biochemical routes, enzyme and substrate specificity, functional site hierarchy, cellular compartmentalization, and physiological roles across immune and disease contexts. Such work will determine whether pyruvylation can be developed into a useful biomarker or therapeutically tractable regulatory axis in infection, inflammation, cancer, and other metabolism-associated disorders.

Whether pyruvylation ultimately joins lactylation as a broadly conserved immunoregulatory mechanism remains to be determined. Nevertheless, its discovery has opened a new avenue for understanding how metabolic states are encoded into immune signaling, providing a foundation for future mechanistic and translational investigation.
